# Management of atrial fibrillation in patients taking targeted cancer therapies

**DOI:** 10.1186/s40959-017-0021-y

**Published:** 2017-03-09

**Authors:** Aarti Asnani, Anastasia Manning, Moussa Mansour, Jeremy Ruskin, Ephraim P. Hochberg, Leon M. Ptaszek

**Affiliations:** 1 Cardio-Oncology Program, Corrigan Minehan Heart Center, Massachusetts General Hospital, 149 13th Street, Room 4.302, Boston, MA 02129 USA; 2 Massachusetts General Hospital, Boston, MA 02129 USA; 3 Cardiac Arrhythmia Service, Corrigan-Minehan Heart Center, Massachusetts General Hospital, 55 Fruit Street, GRB 109, Boston, MA 02114 USA; 4 Hematology/Oncology, Cancer Center, Massachusetts General Hospital, 55 Fruit Street, Boston, MA 02114 USA

**Keywords:** Atrial fibrillation, Cancer, Chemotherapy, Targed therapies, Ibrutinib, Anticoagulation

## Abstract

Atrial fibrillation (AF) is frequently observed in patients being treated for cancer and can lead to increased morbidity and mortality in this population. With the use of newer, targeted cancer therapies, several drug-drug interactions have emerged that complicate the use of antiarrhythmic drugs (AADs) in patients with active malignancy. Moreover, specific targeted therapies such as ibrutinib may contribute directly to the development of AF. The decision to pursue systemic anticoagulation can be challenging in patients with malignancy due to a number of factors, including the need for frequent procedures, the presence of malignancy-related risk factors for bleeding, and limited data regarding the safety of the novel oral anticoagulants (NOACs) in cancer patients. This review describes the challenges associated with AF management in patients with cancer and highlights a number of important drug-drug interactions that can impact patient management.

## Background

AF is commonly diagnosed in the setting of active cancer. Although there are limited epidemiologic data examining the prevalence of AF in this population, one recently published study of over 24,000 patients with newly diagnosed malignancy reported that 2.4% of patients had pre-existing AF at the time of their cancer diagnosis. An additional 1.8% of patients went on to develop new-onset AF after their initial cancer diagnosis [1]. In comparison, the overall prevalence of AF is approximately 1% in the general population [2]. However, this estimate varies significantly with age, and the prevalence of AF has been reported to be as low as 0.1% in adults less than 55 years of age to as high as 9% in patients greater than 80 years of age. The increased prevalence of AF in patients with cancer, compared to that of the general population, thus likely reflects the increased prevalence of AF in patients with advanced age and/or cardiovascular comorbidities. Patients with cancer who had either baseline or new-onset AF were older, with an average age of 72–75 years compared to 60 years in patients who did not develop AF [1]. Moreover, patients with cancer and AF had higher rates of hypertension, myocardial infarction, and heart failure compared to those patients who did not develop AF. These observations reflect the well-defined relationship between AF, age, and cardiovascular comorbidies in the general population. Compared to patients with baseline AF, those with new-onset AF in the context of malignancy had a 2-fold increased risk of thromboembolism and a 6-fold increased risk of heart failure, highlighting the importance of prompt recognition and treatment of AF during cancer treatment. Notably, new-onset AF may in turn increase the risk of subsequent cancer, particularly within the first 3 months following diagnosis of AF [3, 4]. This effect is not well-understood but may be related to shared risk factors, increased detection of malignancies due to increased health care exposure, or to the unmasking of occult GI malignancies by anticoagulation [5].

AF is particularly common after surgical excision of tumors, with rates ranging from 13% to 65% following lung cancer surgery [6, 7]. Presence of AF after tumor excision may also be an important prognostic marker: lung cancer survivors with post-operative AF have a higher morbidity and mortality compared to those who do not experience AF, an effect which persists at 5 years of follow-up [8]. In parallel with the aging of the general population, the number of cancer survivors in the United States is anticipated to grow to nearly 19 million by the year 2024 [9], in large part due to the advent of more effective, targeted cancer therapies. Many of these patients will develop AF during cancer treatment and follow-up, and it will become increasingly important to define management strategies specific to this patient population. Systemic inflammation may play a role in the maintenance of AF, as suggested by prior data demonstrating increased levels of C-reactive protein in patients with AF [10]. Patients who are undergoing treatment for cancer may experience a number of issues that can precipitate arrhythmias, including dehydration, electrolyte disorders, and pain leading to increased sympathetic tone. Mass effect of tumors may also be related to AF occurrences. Expansion of tumor within the thoracic cavity can cause external compression of the left atrium [11] and present with new-onset and sometimes refractory AF. Rarely, primary cardiac tumors or cardiac metastases can precipitate AF [12, 13].

Commonly used cancer treatments may also contribute to the development of atrial fibrillation [14]. In particular, targeted therapies such as the tyrosine kinase inhibitor ibrutinib, may increase the incidence of AF and complicate its treatment due to drug-drug interactions. In this review, we provide an overview of several drug-drug interactions between targeted cancer therapies and antiarrhythmic drugs that have the potential to impact AF management. We also review the limited data pertaining to simultaneous use of oral anticoagulants and targeted cancer therapies.

## Antiarrhythmic drug therapy for AF in patients receiving targeted cancer therapies

As opposed to traditional cytotoxic chemotherapy, targeted therapies have been developed to inhibit specific molecular targets essential to tumor survival and proliferation. Targeted therapies include hormone antagonists, signal transduction inhibitors, gene expression modulators, apoptosis inducers, angiogenesis inhibitors, immunotherapies, and monoclonal antibodies [15]. There are currently over 80 FDA-approved targeted therapies, many of which are tyrosine kinase inhibitors used to treat common malignancies such as breast cancer, lung cancer, leukemia, and lymphoma. Given the rapid development of these agents, with many more in the pipeline, it will be important to understand the interactions between targeted therapies and commonly used cardiovascular medications.

Interactions between targeted therapies and AADs can significantly impact the choice of treatment strategy for AF. Newer cancer therapies are often multitargeted, with a different kinome binding profile and therefore a different pharmacokinetic/adverse effect profile for each (Table 1). Co-administration of targeted cancer therapies and AADs may lead to increased levels of one or both drugs, often due to impaired hepatic cytochrome p450 metabolism or inhibition of P-glycoprotein-mediated transport of the targeted therapy. Furthermore, some targeted therapies increase the propensity for bradycardia and QT prolongation in the setting of AADs. Although some AADs can be used with careful electrocardiographic monitoring and/or dose reduction, others are contraindicated in the setting of specific targeted therapies (Tables 2 and 3). These drug-drug interactions can pose a significant challenge for the management of AF in the setting of active malignancy. In general, amiodarone, dronedarone, and the calcium channel blockers have the potential to interact with nearly all the targeted therapies and often require dose adjustment or discontinution of either the AAD or targeted therapy, depending on the combination of agents utilized. Class IA, IC, and III antiarrhythmics are the most likely to cause QT prolongation when used in combination with targeted therapies. In contrast, Class IB antiarrhythmics, particularly mexiletine, are the least likely to cause significant drug-drug interactions when used with the targeted therapies listed in Tables 2 and 3. Finally, carvedilol, propranolol, and nadolol are more likely to participate in drug-drug interactions than metoprolol, atenolol, and pindolol in the setting of targeted therapies. Even so, use of AV nodal blockers may be limited by bradycardia, hypotension, and other side effects.

**Table 1:** Pharmacokinetics of commonly used targeted cancer therapies

Tyrosine Kinase Inhibitors	Molecular Target(s)	FDA-Approved Indications	Clinically Significant Drug Metabolism/Elimination	Clinically Significant Adverse Effects
Afatinib	ErbB family (EGFR, HER-2,-4)	NSCLC	P-gp substrate	
Axitinib	VEGFR-1,-2,-3	RCC	CYP3A4 substrate	
Bosutinib	BCR-ABL, SRC family (SRC, LYN, HCK), c-KIT, PDGFR	CML	CYP3A4 substrate; P-gp inhibitor	QT prolongation
Cabozantinib	HGFR, RET, VEGFR-1, −2, −3, KIT, FLT3, TIE-2, TRKB, AXL	Medullary thyroid cancer	CYP3A4 substrate	
Ceritinib	ALK, IGF-1R, InsR, ROS1	NSCLC	CYP3A4 substrate & strong inhibitor; P-gp substrate	QT prolongation Bradycardia
Crizotinib	ALK, HGFR, RON	NSCLC	CYP3A4 substrate & moderate inhibitor; CYP2B6 moderate inhibitor; P-gp substrate and inhibitor	QT prolongation Bradycardia
Dasatinib	SRC family (SRC, LCK, YES, FYN), BCR-ABL, c-KIT, EPH-A2, PDGFR-β	ALL, CML	CYP3A4 substrate & weak inhibitor	QT prolongation
Erlotinib	EGFR	NSCLC, Pancreatic cancer	CYP3A4 substrate	
Gefitinib	EGFR	NSCLC	CYP3A4 substrate; CYP2D6 substrate	
Ibrutinib	BTK	CLL, MCL, WM	CYP3A4 substrate; P-gp inhibitor	
Imatinib	BCR-ABL, c-KIT, PDGFR, SCF	ALL, CEL, CML, GIST, ASM, DFSP, MDS/MPD	CYP3A4 substrate & moderate inhibitor; P-gp substrate	
Lapatinib	EGFR, HER2	Breast cancer	CYP3A4 substrate; CYP2C8 moderate inhibitor; P-gp substrate & inhibitor	QT prolongation
Lenvatinib	VEGFR-1,-2,-3, FGFR-1,-2,-3,-4, PDGF-α, KIT, RET	Thyroid cancer		QT prolongation
Nilotinib	BCR-ABL, PDGFR, c-KIT	CML	CYP3A4 substrate & moderate inhibitor; CYP2D6 moderate inhibitor; CYP2C8 moderate inhibitor; P-gp inhibitor	QT prolongation
Osimertinib	EGFR	NSCLC	CYP3A4 substrate; BRCP inhibitor	QT prolongation
Pazopanib	VEGFR-1,-2,-3, PDGFR-α,-β, FGFR-1,-3, c-KIT, IL-2 inducible TcK, Lck, c-Fms	RCC, STS	CYP3A4 substrate; P-gp substrate	QT prolongation
Ponatinib	BCR-ABL, VEGFR, FGFR, PDGFR, EPH, SRC, KIT, RET, TIE2, FLT3	ALL, CML	CYP3A4 substrate	
Regorafenib	VEGFR-1,-2,-3, KIT, PDGFR-α,-β, RET, FGFR-1,-2, TIES2, DDR2, TrkA, EPH-A2, RAF-1, BRAF, BRAFV600E, SAPK2, PTK5, ABL	Colorectal cancer, GIST	CYP3A4 substrate	Bradycardia
Ruxolitinib	JAK-1,-2	Myelofibrosis, Polycythemia vera	CYP3A4 substrate	Bradycardia
Sorafenib	RAF, VEGFR-1,-2,-3, PDGFR-β, c-KIT, FLT-3, RET, RET/PTC	RCC, HCC, Thyroid carcinoma	CYP2C9 moderate inhibitor; CYP2B6 moderate inhibitor	QT prolongation
Sunitinib	PDGFR-α,-β, VEGFR-1,-2,-3, FLT3, CSF-1R, RET	RCC, GIST, PNET	CYP3A4 substrate; P-gp inhibitor	QT prolongation
Vandetanib	EGFR, VEGFR, RET, BRK, TIES2, EPH, SRC	Medullary thyroid cancer	P-gp inhibitor	QT prolongation

*ALK* anaplastic lymphoma kinase, *BCR-ABL* fusion gene, *BRAF* B-Raf Proto-Oncogene, Serine/Threonine Kinase, *BRK* protein tyrosine kinase 6, *BTK* Bruton’s Tyrosine Kinase, *c-Fms* transmembrane glycoprotein receptor tyrosine kinase, *c-KIT* cytokine receptor, *CSF-1R* colony-stimulating factor type 1, *DDR2* discoidin domain receptor 2, *EGFR* epidermal growth factor receptor, *EPH* Ephrin Receptor, *ErbB* family of tyrosine kinase inhibitors, *FGFR* fibroblast growth factor receptor, *FLT* FMS-like Tyrosine Kinase, *HCK* Hemopoietic cell kinase, *HER* human epidermal growth factor receptor, *HGFR* hepatocyte growth factor receptor, *IGF-1R* insulin-like growth factor 1 receptor. IL-2 inducable, *TCK* interleukin-2 receptor inducible T-cell Kinase, *InsR* insulin receptor, *JAK* Janus Associated Kinases, *Lck* Leukocyte-specific Protein Tyrosine Kinase, *PDGFR* platelet-derived growth factor receptor, *PTK* protein tyrosine kinase 2 beta, *RET* “Rearranged During Transfection”, *RON* Recepteur d’Origine Nantais, *SCF* stem cell factor, *TIE* Tyrosine Kinase with Immunoglobulin-like and EGF-like Domains, *VEGFR* vascular endothelial growth receptor, *ALL* acute lymphoblastic leukemia, *ASM* aggressive systemic mastocytosis, *CEL* chronic eosinophilic leukemia, *CML* chronic myeloid leukemia, *DFSP* Dermatofibrosarcoma Protuberans, *GIST* gastrointestinal stromal tumor, *HCC* hepatocellular carcinoma, *MCL* mantle cell lymphoma, *MDS/MPD* myelodysplastic syndrome/myeloproliferative disease, *NSCLC* non-small cell lung cancer, *PNET* pancreatic neuroendocrine tumor, *RCC* renal cell carcinoma, *STS* soft tissue sarcoma, *WM* Waldenstrom’s Macroglobulinemia, *BRCP* breast cancer resistance protein, *P-gp* P-glycoprotein

**Table 2:** Drug-drug interactions and predicted plasma levels of antiarrhythmics and targeted cancer therapies

		Anti-arrhythmics																					
	Vaughan Williams Class	1a			1b		1c		2							3					4		Misc.
		Quin-idine	Diso-pyra-mide	Procai-namide	Lido-caine	Mexil-etine	Flec-ainide	Propa-fenone	Meto-prolol	Aten-olol	Carve-dilol	Labe-talol	Propr-anolol	Nad-olol	Pind-olol	Sotalol	Dofet-ilide	Ibut-ilide	Amio-darone	Drone-darone	Diltia-zem	Verap-amil	Digo-xin
Tyrosine Kinase Inhibitors	Afatinib	*TA* ^D^									*TA* ^D^		*T* ^D^	A					*TA* ^D^	*T* ^D^	B	*TA* ^D^	B
	Axitinib																			T*	T*	T*	
	Bosutinib	**T**	*Q*	*Q*			Q	Q			**T**		**T**	A		*Q*	*Q*	*Q*	**T**	**T**	**T**	**T**	A
	Cabozantinib	A									A			A					TA	T*	T*	T*	A
	Ceritinib	**Q**	**Q**	**Q**	*T*		*Q*	**B/Q**	**B**	**B**	**B**	**B**	**B**	**B**	**B**	**B/Q**	**Q**	**Q**	**B/Q**	A/B/Q*	B*	B*	**B**
	Crizotinib	**Q**	**Q**	**Q**	A		*Q*	*A/Q*	B	B	TA/B	B	TA/B	A/B	B	**Q**	**Q**	**Q**	**Q**	**Q**	TA/B	TA/B	A/B
	Dasatinib	*A/Q*	*A/Q*	*Q*	A		Q	Q			A		A			*Q*	*A/Q*	*Q*	*A/Q*	TA/Q*	TA*	TA*	A
	Erlotinib																		*T/D*	T*	T*	*T* ^D^	
	Gefitinib	*T*							*A*										T	T	T	T	
	Ibrutinib	A									A			A					TA	**T/D**	**TA/D**	TA*	A
	Imatinib	TA	A		A			A	*A*		TA		TA	A			A		TA	TA	TA	TA	A
	Lapatinib	*TA/Q*	*Q*	*Q*			Q	Q			TA		T	A		*Q*	*A/Q*	*Q*	*TA/Q*	T/Q*	TA*	TA*	A
	Lenvatinib	**Q**	**Q**	**Q**			*Q*	*Q*			T		T			**Q**	**Q**	**Q**	**Q**	**Q**	T	T	
	Nilotinib	**Q**	**Q**	**Q**	A	A	**Q**	**Q**	*A*		TA		TA	A	A	**Q**	**Q**	**Q**	**Q**	Q*	TA*	TA*	A
	Osimertinib	**Q**	**Q**	**Q**	A	A	*A/Q*	*A/Q*			TA		TA			**Q**	**Q**	**Q**	**Q**	**Q**	TA	TA	A
	Pazopanib	**T/Q**	**Q**	**Q**			*Q*	*Q*	*A*		**T**		**T**			**Q**	**Q**	**Q**	**T/Q**	T/Q*	T*	T*	
	Ponatinib	T/A									TA		T	A					TA	T*	TA*	TA*	A
	Regorafenib	A							B	B	A/B	B	B	A/B	B	B			A/B	T/B	TA/B	TA/B	A/B
	Ruxolitinib							B	B	B	B	B	B	B	B	B			B	T/B*	T/B*	T/B*	B
	Sorafenib	*A/Q*	*Q*	*Q*	A		Q	Q			A					*Q*	*Q*	*Q*	*Q*	*T/Q*	TA	TA	
	Sunitinib	*A/Q*	*Q*	*Q*			Q	Q			A			A		*Q*	*Q*	*Q*	*A/Q*	T/Q*	TA*	TA*	A
	Vandetanib	**Q**	**Q**	**Q**			**Q**	**Q**			A			A		**Q**	**Q**	**Q**	**Q**	**Q**	TA	TA	A

Drug-drug interactions are predicted based on pharmacokinetics, given the limited data available from patient studies

Underlined = "Alter combination" Bold = "Combination is contraindicated"

**Table 3:** Recommended dose adjustments in the setting of drug-drug interactions

TKI	Interaction	Dose Adjustments
Nilotinib	*	Should interrupt TKI therapy. If cannot interrupt TKI therapy, consider reducing TKI dose to: 300 mg QD (Resistant/intolerant Ph + CML) 200 mg QD (Newly diagnosed Chronic Phase Ph + CML, with careful monitoring, especially of QT interval) If 3A4 inhibitor discontinued, allow washout period prior to uptitrating dose.
Pazopanib	*	Reduce TKI dose to 400 mg QD (careful monitoring). Further dose reductions may be necessary if toxicity occurs.
Ponatinib	*	Reduce TKI dose to 30 mg QD
Ruxolitinib	*	Dose of TKI: Myelofibrosis -Platelets ≥ 100,000/mm^3^: 10 mg BID -Platelets 50,000/mm^3^ - 100,000/mm^3^: 5 mg QD Polycythemia Vera: 5 mg BID Patients on already stable TKI doses of: -5 mg QD: AVOID or interrupt TKI therapy -5 mg BID: Reduce TKI dose to 5 mg QD - ≥ 10 mg BID: Reduce TKI dose by 50% (rounded to closest available tablet strength)
	D (Canadian Labeling)	Reduce TKI dose by 50% (rounded to closest available tablet strength). Monitor hematologic parameters more frequently (i.e. twice weekly). -If platelets < 100,000/mm3: AVOID -Titrate dose based on safety & efficacy
Sunitinib	*	Consider TKI dose reduction to minimum of: GIST, RCC: 37.5 mg/day PNET: 25 mg/day

## Ibrutinib and AF

Recent clinical trials have demonstrated particularly high rates of atrial fibrillation with the targeted therapy ibrutinib, a covalent, irreversible inhibitor of Bruton’s tyrosine kinase (BTK). Ibrutinib is currently FDA-approved for the treatment of relapsed or refractory chronic lymphocytic leukemia (CLL) and mantle cell lymphoma as well as Waldenström macroglobulinemia. In the initial clinical trials of ibrutinib, serial electrocardiographic studies were performed without any evidence of arrhythmia [16, 17]. Ibrutinib was otherwise found to be well-tolerated, with only mild-moderate GI side effects and no significant bone marrow suppression or life-threatening infections [18, 19]. However, in the RESONATE trial, which included 391 patients with relapsed or refractory CLL or small lymphocytic lymphoma, 3% of patients on ibrutinib developed grade 3 (severe) atrial fibrillation, as opposed to 0% of patients in the ofatumumab-treated standard therapy group [20]. The development of atrial fibrillation required cessation of therapy in one patient. Furthermore, an additional four patients in the ibrutinib group experienced grade 1 or 2 (mild or moderate) atrial fibrillation, compared to one patient in the ofatumumab group.

Similar observations were made in a subsequent small, open-label, Phase 2 clinical trial studying ibrutinib in combination with rituximab in 50 patients with relapsed or refractory mantle cell lymphoma [21]. In this study, six patients (12%) developed grade 3 atrial fibrillation, and three patients required discontinuation of ibrutinib for atrial fibrillation. An additional patient developed mild to moderate atrial fibrillation and was continued on the study regimen. Given the demographics of the study population, the authors postulated that advanced age (median 67 years) and prior exposure to anthracyclines (at least 24% of patients) may have contributed to the increased rate of arrhythmia observed in the trial.

Based on these data, an international retrospective study was performed to define the clinical characteristics of patients treated for CLL who developed AF on ibrutinib [22]. In this study, which included 56 patients, AF onset generally occurred between 3 and 8 months after initiation of ibrutinib, and 76% of patients developed AF during the first year of ibrutinib therapy. The majority of patients were treated with AADs or electrical cardioversion. Three patients experienced congestive heart failure while in AF, and one patient had an ischemic stroke. Of note, only 48% of patients received oral anticoagulation. This was likely due to the concern for thrombocytopenia and significant bleeding events, the latter of which occurred in 14% of patients. Ibrutinib was either stopped or dose-reduced in 35 patients, and it was continued at full dose in 21 patients. Interestingly, in four patients ibrutinib therapy was stopped and then restarted at full dose. All of these patients were then able to continue treatment for the remainder of the study, suggesting that re-challenge of ibrutinib may be successful once effective therapies have been initiated for AF. Ibrutinib has significant intrinsic anticoagulant activity due to unclear mechanisms. In a small series, up to 55% of patients treated with ibrutinib suffered from mild to moderate bleeding events during the course of their therapy [23]. Because of this anticoagulant activity, additional platelet inhibition is generally contraindicated during the ibrutinib therapy course.

These clinical observations have prompted preliminary investigations into the molecular mechanisms contributing to ibrutinib-mediated atrial fibrillation. For many of the novel targeted cancer therapies, both on-target and off-target toxicities have been observed. Although one report suggests that ibrutinib-induced atrial fibrillation may be related to on-target toxicity via inhibition of BTK [24], newer BTK inhibitors such as acalabrutinib have not been associated with increased rates of AF [25], suggesting an off-target mechanism. Elucidation of the molecular mechanisms underlying this process will become increasingly important as clinical indications for the use of ibrutinib and other BTK inhibitors continue to expand over time.

## Anticoagulation for AF in patients with cancer

The decision to initiate therapeutic anticoagulation in patients with active cancer can be challenging. Malignancy increases both the risk of thromboembolic events as well as the risk of bleeding, particularly in the setting of thrombocytopenia and intracranial disease. The choice of cancer therapy may influence the decision to anticoagulate, as some targeted therapies such as angiogenesis inhibitors specifically increase the risk of thromboembolism [26]. Traditional risk stratification tools to evaluate the thromboembolic risk associated with AF may not apply in patients with active cancer. In patients with a new diagnosis of cancer, an increasing CHADS_2_ score was predictive of thromboembolism only in patients with baseline AF and not in those patients with new-onset AF, though it did correlate with increasing mortality in the latter population [1]. The more contemporary CHA_2_DS_2_-VASc score, as well as the HAS-BLED score to evaluate the bleeding risk associated with therapeutic anticoagulation, have not been validated in patients with active malignancy.

Vitamin K antagonists (VKAs) such as warfarin have long been the gold standard for anticoagulation to minimize stroke risk in the setting of atrial fibrillation. However, several issues related to cancer and its treatment can complicate warfarin therapy, including drug-drug interactions when co-administered with targeted cancer treatments (Table 4) and changes in nutritional status related to nausea/vomiting, anorexia, and weight loss [27]. In addition, cancer patients are more likely to interrupt anticoagulation for surgeries and other invasive procedures, as well as for thrombocytopenia. One retrospective, non-randomized study followed 2168 consecutive patients with non-valvular AF and newly diagnosed malignancy for an average of nearly 4 years [28]. Patients were propensity-matched and outcomes assessed based on the use of VKAs for oral anticoagulation. No significant difference was observed in the composite endpoint of major adverse cardiac events (MACE; ischemic stroke, myocardial infarction, pulmonary thromboembolism) or major bleeding in patients treated with VKAs when compared to those who were not anticoagulated. However, only 12% of patients on VKA therapy achieved a target INR of 2.0-3.0, and difficulty maintaining therapeutic anticoagulation likely contributed to the observed lack of benefit. Interestingly, patients who maintained a target therapeutic range (TTR) greater than 60% and did not experience a MACE within the first year of their cancer diagnosis demonstrated an improved cumulative survival free of the composite endpoint compared to those patients who had not been anticoagulated. These observations suggest that VKAs such as warfarin may be less effective due to the difficulty in achieving target INR values during active treatment for cancer. Those patients who are able to maintain therapeutic levels of warfarin may benefit from anticoagulation, although larger randomized trials will be necessary to assess the risk/benefit ratio in this patient population.

**Table 4:** Drug-drug interactions and predicted plasma levels of oral anticoagulants and targeted cancer therapies

		Oral Anticoagulants				
		Warfarin	Dabigatran	Rivaroxaban	Apixaban	Edoxaban
Tyrosine Kinase Inhibitors	Afatinib					
	Axitinib					
	Bosutinib					
	Cabozantinib					
	Ceritinib	*↑OAC levels*		*↑OAC levels*	*↑OAC levels*	
	Crizotinib	↑OAC levels	↑OAC levels*	↑OAC levels*	↑OAC levels*	↑OAC levels*
	Dasatinib	↑OAC levels & effect	↑OAC effect	↑OAC levels & effect	↑OAC levels & effect	↑OAC effect
	Erlotinib	↑OAC levels				
	Gefitinib	↑OAC effect				
	Ibrutinib	↑OAC effect	↑OAC levels & effect*	↑OAC levels & effect*	↑OAC levels & effect*	↑OAC levels & effect*
	Imatinib	*↑OAC levels & effect*		↑OAC levels	↑OAC levels	
	Lapatinib		↑OAC levels*	↑OAC levels*	↑OAC levels*	↑OAC levels*
	Lenvatinib					
	Nilotinib	↑OAC levels	↑OAC levels*	↑OAC levels*	↑OAC levels*	↑OAC levels*
	Osimertinib	↑OAC levels		↑OAC levels	↑OAC levels	
	Pazopanib					
	Ponatinib					
	Regorafenib	↑OAC effect				
	Ruxolitinib					
	Sorafenib	*↑OAC levels & effect*			↑OAC levels	
	Sunitinib		↑OAC levels*	↑OAC levels*	↑OAC levels*	↑OAC levels*
	Vandetanib		↑OAC levels*	↑OAC levels*	↑OAC levels*	↑OAC levels*

Drug-drug interactions are predicted based on pharmacokinetics, given the limited data available from patient studies

Underlined = "Alter combination"

Given the limitations of VKA therapy, alternative agents have been proposed for anticoagulation in the setting of AF and cancer. The use of low-molecular-weight heparin has been advocated for the treatment of venous thromboembolism in patients with active cancer [29], although its use in AF has not been well-studied. The NOACs (novel oral anticoagulants; dabigatran, rivaroxaban, apixaban, and edoxaban) are appealing alternatives, as they do not require frequent laboratory monitoring. Outcomes associated with NOAC use in patients with cancer are not well-studied as patients with active cancer were excluded from all major clinical trials studying the use of these agents in AF [30–33]. Although NOAC trials for VTE treatment included patients with cancer, the total number of cancer-affected patients was too small (3–9%) to allow for definitive conclusions [28]. Acute fluctuations in renal and hepatic function can affect plasma levels of NOACs and may require dose adjustment or discontinuation of these agents. Notably, P-glycoprotein (P-gp)-mediated transport is important for NOAC elimination [34] and can be affected by a number of targeted cancer therapies (Table 4). These drug-drug interactions may result in increased NOAC distribution to the brain and potentially increase the risk of intracranial bleeding, although there is not enough data in the literature to allow for an accurate quantification of relative risk. For instance, although the precursor drug dabigatran etexilate is a P-gp substrate, dabigatran itself is not [35]. The effect of coadministration of P-gp inhibitors and inducers on dabigatran levels in the brain and the related bleeding risk have not been directly assessed in either preclinical models or in patients. With regard to rivaroxaban, knockout of P-gp in mice resulted in a mild increase in drug concentrations in the brain [36]. However, it is not clear whether these observations extend to patients co-treated with rivaroxaban and P-gp modulators. Although apixaban is a P-gp substrate [37], it appears to have limited penetration across the blood-brain barrier [38]. Thus, consideration of NOAC treatment in patients with AF undergoing active treatment for cancer should take into account the pharmacokinetic properties of targeted cancer therapies when applicable, although data supporting the clinical relevance of these drug-drug interactions remain limited.

## Conclusions

The development of novel targeted cancer therapies has improved patient survival and increased the tolerability of treatment regimens for a number of different malignancies. Atrial fibrillation has emerged as an important cardiovascular side effect in the setting of malignancy, particularly with the use of ibrutinib, which may directly contribute to the pathogenesis and maintenance of AF. Management of AF in cancer patients can be challenging given the many drug-drug interactions between targeted therapies and AADs. Moreover, recognition and treatment of atrial fibrillation in this setting will be critical to minimize adverse events such as stroke, although the choice of anticoagulation strategy in these patients remains challenging and requires further study. In light of the improved toxicity profile of targeted therapies, many patients are now staying on these medications for long-term cancer suppression. In the case of ibrutinib, a recent study highlighted the durability of response in patients with mantle cell lymphoma, 20% of whom continued treatment for over 2 years [33]. Atrial fibrillation was observed in 11% of patients in this study, leading to dose reduction of the study drug in one patient. As the use of ibrutinib and other targeted cancer therapies continues to expand, a multidisciplinary approach including cardiology and oncology will be important in minimizing the morbidity and mortality associated with AF in the setting of active malignancy.

## Abbreviations

AADs: Antiarrhythmic drugs
AF: Atrial fibrillation
BTK: Bruton’s tyrosine kinase
CLL: Chronic lymphocytic leukemia
MACE: Major adverse cardiac events
NOACs: Novel oral anticoagulants
P-gp: P-glycoprotein
TTR: Target therapeutic range
VKAs: Vitamin K antagonists

## References

[1] Hu YF, Liu CJ, Chang PM, Tsao HM, Lin YJ, Chang SL, Lo LW, Tuan TC, Li CH, Chao TF (2013). Incident thromboembolism and heart failure associated with new-onset atrial fibrillation in cancer patients. Int J Cardiol.

[2] Go AS, Hylek EM, Phillips KA, Chang Y, Henault LE, Selby JV, Singer DE (2001). Prevalence of diagnosed atrial fibrillation in adults: national implications for rhythm management and stroke prevention: the AnTicoagulation and Risk Factors in Atrial Fibrillation (ATRIA) Study. JAMA.

[3] Ostenfeld EB, Erichsen R, Pedersen L, Farkas DK, Weiss NS, Sorensen HT (2014). Atrial fibrillation as a marker of occult cancer. PLoS One.

[4] Conen D, Wong JA, Sandhu RK, Cook NR, Lee IM, Buring JE, Albert CM (2016). Risk of malignant cancer among women with New-onset atrial fibrillation. JAMA Cardiol.

[5] Rahman F, Ko D, Benjamin EJ (2016). Association of atrial fibrillation and cancer. JAMA Cardiol.

[6] Onaitis M, D’Amico T, Zhao Y, O’Brien S, Harpole D (2010). Risk factors for atrial fibrillation after lung cancer surgery: analysis of the Society of Thoracic Surgeons general thoracic surgery database. Ann Thorac Surg.

[7] Nojiri T, Inoue M, Maeda H, Takeuchi Y, Sawabata N, Shintani Y, Yamamoto K, Okumura M (2013). Low-dose human atrial natriuretic peptide for the prevention of postoperative cardiopulmonary complications in chronic obstructive pulmonary disease patients undergoing lung cancer surgery. Eur J Cardiothorac Surg.

[8] Imperatori A, Mariscalco G, Riganti G, Rotolo N, Conti V, Dominioni L (2012). Atrial fibrillation after pulmonary lobectomy for lung cancer affects long-term survival in a prospective single-center study. J Cardiothorac Surg.

[9] DeSantis CE, Lin CC, Mariotto AB, Siegel RL, Stein KD, Kramer JL, Alteri R, Robbins AS, Jemal A (2014). Cancer treatment and survivorship statistics, 2014. CA Cancer J Clin.

[10] Chung MK, Martin DO, Sprecher D, Wazni O, Kanderian A, Carnes CA, Bauer JA, Tchou PJ, Niebauer MJ, Natale A, Van Wagoner DR (2001). C-reactive protein elevation in patients with atrial arrhythmias: inflammatory mechanisms and persistence of atrial fibrillation. Circulation.

[11] Bayraktar UD, Dufresne A, Bayraktar S, Purcell RR, Ajah OI (2008). Esophageal cancer presenting with atrial fibrillation: a case report. J Med Case Rep.

[12] Holzhauser L, Heymer J, Kasner M, Landmesser U, Skurk C (2015). Rare case of a multilocular primary cardiac intimal sarcoma presenting as left atrial mass with new onset atrial fibrillation. Eur Heart J.

[13] Cohen JE, Kogan J, Oren S, Mazza M (2011). Primary cardiac lymphoma presenting with atrial fibrillation. Isr Med Assoc J.

[14] Guglin M, Aljayeh M, Saiyad S, Ali R, Curtis AB (2009). Introducing a new entity: chemotherapy-induced arrhythmia. Europace.

[15] National Cancer Institute, [https://www.cancer.gov/about-cancer/treatment/types/targeted-therapies/targeted-therapies-fact-sheet]. Accessed 24 Jan 2017.

[16] Byrd JC, O’Brien S, James DF (2013). Ibrutinib in relapsed chronic lymphocytic leukemia. N Engl J Med.

[17] Advani RH, Buggy JJ, Sharman JP, Smith SM, Boyd TE, Grant B, Kolibaba KS, Furman RR, Rodriguez S, Chang BY (2013). Bruton tyrosine kinase inhibitor ibrutinib (PCI-32765) has significant activity in patients with relapsed/refractory B-cell malignancies. J Clin Oncol.

[18] Burger JA, Keating MJ, Wierda WG, Hartmann E, Hoellenriegel J, Rosin NY, de Weerdt I, Jeyakumar G, Ferrajoli A, Cardenas-Turanzas M (2014). Safety and activity of ibrutinib plus rituximab for patients with high-risk chronic lymphocytic leukaemia: a single-arm, phase 2 study. Lancet Oncol.

[19] O’Brien S, Furman RR, Coutre SE, Sharman JP, Burger JA, Blum KA, Grant B, Richards DA, Coleman M, Wierda WG (2014). Ibrutinib as initial therapy for elderly patients with chronic lymphocytic leukaemia or small lymphocytic lymphoma: an open-label, multicentre, phase 1b/2 trial. Lancet Oncol.

[20] Byrd JC, Brown JR, O’Brien S, Barrientos JC, Kay NE, Reddy NM, Coutre S, Tam CS, Mulligan SP, Jaeger U (2014). Ibrutinib versus ofatumumab in previously treated chronic lymphoid leukemia. N Engl J Med.

[21] Wang ML, Lee H, Chuang H, Wagner-Bartak N, Hagemeister F, Westin J, Fayad L, Samaniego F, Turturro F, Oki Y, et al. Ibrutinib in combination with rituximab in relapsed or refractory mantle cell lymphoma: a single-centre, open-label, phase 2 trial. Lancet Oncol. 2016;17(1):48–56. 10.1016/S1470-2045(15)00438-6 26640039

[22] Thompson PA, Levy V, Tam CS, Al Nawakil C, Goudot FX, Quinquenel A, Ysebaert L, Michallet AS, Dilhuydy MS, Van Den Neste E (2016). Atrial fibrillation in CLL patients treated with ibrutinib. An international retrospective study. Br J Haematol.

[23] Lipsky AH, Farooqui MZ, Tian X, Martyr S, Cullinane AM, Nghiem K, Sun C, Valdez J, Niemann CU, Herman SE (2015). Incidence and risk factors of bleeding-related adverse events in patients with chronic lymphocytic leukemia treated with ibrutinib. Haematologica.

[24] McMullen JR, Boey EJ, Ooi JY, Seymour JF, Keating MJ, Tam CS (2014). Ibrutinib increases the risk of atrial fibrillation, potentially through inhibition of cardiac PI3K-Akt signaling. Blood.

[25] Byrd JC, Harrington B, O’Brien S, Jones JA, Schuh A, Devereux S, Chaves J, Wierda WG, Awan FT, Brown JR (2016). Acalabrutinib (ACP-196) in relapsed chronic lymphocytic leukemia. N Engl J Med.

[26] Choueiri TK, Schutz FA, Je Y, Rosenberg JE, Bellmunt J (2010). Risk of arterial thromboembolic events with sunitinib and sorafenib: a systematic review and meta-analysis of clinical trials. J Clin Oncol.

[27] Rose AJ, Sharman JP, Ozonoff A, Henault LE, Hylek EM (2007). Effectiveness of warfarin among patients with cancer. J Gen Intern Med.

[28] Lee YJ, Park JK, Uhm JS, Kim JY, Pak HN, Lee MH, Sung JH, Joung B (2016). Bleeding risk and major adverse events in patients with cancer on oral anticoagulation therapy. Int J Cardiol.

[29] Lee AY, Levine MN, Baker RI, Bowden C, Kakkar AK, Prins M, Rickles FR, Julian JA, Haley S, Kovacs MJ (2003). Low-molecular-weight heparin versus a coumarin for the prevention of recurrent venous thromboembolism in patients with cancer. N Engl J Med.

[30] Connolly SJ, Ezekowitz MD, Yusuf S, Eikelboom J, Oldgren J, Parekh A, Pogue J, Reilly PA, Themeles E, Varrone J (2009). Dabigatran versus warfarin in patients with atrial fibrillation. N Engl J Med.

[31] Patel MR, Mahaffey KW, Garg J, Pan G, Singer DE, Hacke W, Breithardt G, Halperin JL, Hankey GJ, Piccini JP (2011). Rivaroxaban versus warfarin in nonvalvular atrial fibrillation. N Engl J Med.

[32] Granger CB, Alexander JH, McMurray JJ, Lopes RD, Hylek EM, Hanna M, Al-Khalidi HR, Ansell J, Atar D, Avezum A (2011). Apixaban versus warfarin in patients with atrial fibrillation. N Engl J Med.

[33] Giugliano RP, Ruff CT, Braunwald E, Murphy SA, Wiviott SD, Halperin JL, Waldo AL, Ezekowitz MD, Weitz JI, Spinar J (2013). Edoxaban versus warfarin in patients with atrial fibrillation. N Engl J Med.

[34] Wessler JD, Grip LT, Mendell J, Giugliano RP (2013). The P-glycoprotein transport system and cardiovascular drugs. J Am Coll Cardiol.

[35] Stollberger C, Finsterer J (2015). Relevance of P-glycoprotein in stroke prevention with dabigatran, rivaroxaban, and apixaban. Herz.

[36] Gnoth MJ, Buetehorn U, Muenster U, Schwarz T, Sandmann S (2011). In vitro and in vivo P-glycoprotein transport characteristics of rivaroxaban. J Pharmacol Exp Ther.

[37] Zhang D, He K, Herbst JJ, Kolb J, Shou W, Wang L, Balimane PV, Han YH, Gan J, Frost CE, Humphreys WG (2013). Characterization of efflux transporters involved in distribution and disposition of apixaban. Drug Metab Dispos.

[38] Wang L, He K, Maxwell B, Grossman SJ, Tremaine LM, Humphreys WG, Zhang D (2011). Tissue distribution and elimination of [14C]apixaban in rats. Drug Metab Dispos.

